# Neutralization Effect of Sera against Delta and Omicron in Patients Recovering from COVID-19 and Inactivated Vaccine Recipients

**DOI:** 10.3390/vaccines11020471

**Published:** 2023-02-17

**Authors:** Yajuan Zhu, Qianhong Zhong, Zhanzhong Ma, Shuang Liu, Yunhua Lan, Bo Peng, Xiaomin Zhang, Xiaolu Shi, Jing Qu, Zhilong Wu, Zhimeng Zhao, Xilin Zhang, Dingmei Zhang

**Affiliations:** 1 Department of Epidemiology, School of Public Health, Sun Yat-Sen University, Guangzhou 510080, China; 2 Department of Tuberculosis Prevention and Control, Foshan Fourth People’s Hospital, Foshan 528208, China; 3 Prenatal Diagnosis Center, Yuebei People’s Hospital of Shantou University Medical College, Shaoguan 512027, China; 4 Institute of Immunization Program, Guangdong Provincial Center for Disease Control and Prevention, Guangzhou 511430, China; 5 Department of Pathogen Biology, Shenzhen Center for Disease Control and Prevention, Shenzhen 518055, China; 6 NMPA Key Laboratory for Quality Monitoring and Evaluation of Vaccines and Biological Products, Guangzhou 510080, China

**Keywords:** COVID-19, inactivated vaccine, Delta, Omicron

## Abstract

This study aims to analyze the serum neutralization capacity against Delta and Omicron variants in three clusters of individuals, including those who had recovered from COVID-19 and those who had received two and three doses of inactivated vaccine. Pseudovirus neutralization tests were performed on serum samples. The neutralizing titers between different groups were compared using the Wilcoxon’s signed-rank test. Among the two-dose vaccinees, the neutralization titers of the Omicron variant were reduced by approximately 3.1-fold compared to the wild-type virus (*p* < 0.05). Meanwhile, among the three-dose vaccinees, the neutralization titers for Delta and Omicron variants were 3.5-fold (*p* < 0.05) and 5.0-fold (*p* < 0.05) lower, respectively, as compared to the wild-type virus. In addition, among the recovering patients, the neutralization titers for Delta and Omicron variants were 3.9-fold (*p* < 0.05) and 29.1-fold (*p* < 0.05) lower, respectively, as compared to the wild-type virus. Overall, only 12.0% (11/92) of participants showed neutralizing titers against Omicron above the detection limit. The ability to neutralize wild-type pseudovirus was significantly boosted in three-dose vaccinees as compared to two-dose vaccinees. Sera from recovered patients showed greater neutralizing titers for the wild-type and Delta pseudoviruses than the two- and three-dose inactivated vaccine groups. The present study revealed a loss of neutralizing activity against the Omicron variant in almost all samples. Moreover, the immunization effect obtained through natural infection is more robust than that from the active immunization method of vaccination.

## 1. Introduction

Coronavirus disease (COVID-19), caused by severe acute respiratory syndrome coronavirus 2 (SARS-CoV-2), is a pandemic of international concern and has persisted to the present day, thereby posing a huge threat to human life and general well-being. As of 7 October 2022, the World Health Organization (WHO) has reported more than 617 million cases and 6 million deaths related to COVID-19 [1]. Therefore, SARS-CoV-2 has a high variability profile. In addition, as of 4 October 2022, WHO has identified the following five variants of concern (VOCs): Alpha, Beta, Gamma, Delta, and Omicron [2]. Moreover, these variant strains are associated with one or more changes in global public health significance through comparative assessments. The highly transmissible Delta variant emerged between October and December 2020, and its emergence threatened worldwide efforts to control the pandemic. Meanwhile, the Omicron variant was initially reported in South Africa in November 2021, and is spreading rapidly in various countries worldwide with greater transmission than any previous variants [3,4]. Currently, given its high transmission and immune escape capacity, Omicron is the leading and the most severely mutated variant to date worldwide. Furthermore, the Omicron variant has acquired approximately 60 amino acid site mutations, and the spike protein has more than 30 mutated sites as compared to the original SARS-CoV-2 isolate in 2019. Omicron is over 10 times more infectious than the wild-type virus and approximately 2.8 times more infectious than the Delta variant. In addition, Omicron is associated with immune escape and may have an 88% probability of escaping current vaccines [3].

Given the lack of specific antiviral drugs and effective treatment, COVID-19 vaccination has been proven to be the most effective method of preventing SARS-CoV-2 infection, severe illness, and death. The continued mutation of SARS-CoV-2, particularly the recent appearance of the Omicron and Delta variants, has raised public health concerns about the effectiveness of the vaccine. Therefore, booster doses have been administered in various countries to improve vaccine immunity. Inactivated vaccines are a classical form of vaccine, and possess excellent safety and efficacy profiles. Furthermore, inactivated vaccines are convenient for long-term storage and transport at conventional refrigerator temperatures (2–8 °C). Consequently, they are suitable for use used particularly in places with limited refrigeration capacity and in developing countries. The BBIBP-CorV vaccine is an inactivated COVID-19 vaccine prepared from *β-propionolactone*, which was first approved for emergency use in 2020. In addition, CoronaVac, an inactivated COVID-19 vaccine with alum as an adjuvant, is a second Chinese vaccine on the WHO COVID-19 vaccine emergency use list.

Schmidt et al. [5] found that serum from COVID-19-recovered individuals had only 1/60th–1/30th of the neutralizing titer for Omicron of that found for the wild strain, and neutralization of Omicron using recovery serum was poor [6]. Moreover, Zhang Wenhong et al. [7] revealed that two-dose vaccinees of inactivated vaccines showed at least a 5.3-fold decrease in antibody titer levels against Omicron as compared to the wild strain, with a significant increase in antibody titers after the third dose. Meanwhile, a study [8] that recruited recipients of three doses of inactivated COVID-19 vaccine determined the neutralizing activity of 99.5%, 98.5%, and 95.5% for the pseudotyped wild-type, Delta, and Omicron variants, respectively. Parallel comparisons of the Omicron and Delta variants with natural infection- or vaccine-triggered neutralization are imperative. Therefore, to provide more comprehensive insights, the present study aims to analyze the serum neutralization capacity against Delta and Omicron variants in three clusters of individuals including those who had recovered from COVID-19, and who had received two and three doses of inactivated vaccine (CoronaVac and BBIBP-CorV). Neutralizing antibody titers are quantified using pseudovirus neutralization tests with wild-type SARS-CoV-2, Delta, and Omicron variants. In addition, the pseudovirus cannot autonomously replicate and is highly safe.

## 2. Materials and Methods

### 2.1. Study Design

This is a descriptive study, and a simple random sample was used as the sampling method. The neutralizing antibody titers were assessed using an in vitro cytological assay. The study was conducted from June 2020 to February 2022 in Guangdong Province, China. Herein, serum samples were collected from participants who received two or three doses of inactivated vaccine at the Foshan Fourth People’s Hospital and Yuebei People’s Hospital. The exclusion criterion was a history of previous SARS-CoV-2 infection. In addition, there were 16 serum samples from recovered patients who were the first wave of patients infected with the original wild-type virus at the beginning of the pandemic in 2020, which were also collected from the Fourth People’s Hospital of Foshan. This study was approved by the Ethics Committee of Sun Yat-sen University.

### 2.2. Pseudovirus Preparation

Wild-type, B.1.617.2 (Delta), and B.1.1.529 (Omicron) variants of SARS-CoV-2 pseudovirus were prepared by Shanghai Jiman Biologicals based on the human immunodeficiency virus (HIV) lentiviral vector and SARS-CoV-2 S protein. Herein, the SARS-CoV-2 S protein was replaced with lentiviral envelope protein VSVG and cotransfected with lentiviral packaging and CMV-GFP-T2A-Luciferase plasmids in 293T cells. Then, the packaging of the SARS-CoV-2 S pseudovirus expressed the SARS-CoV-2 S protein on its surface and carried both GFP fluorescence and Luciferase reporter genes. The activity of the pseudovirus in infected cells can be evaluated by observing the fluorescence and detecting the luciferase activity. However, the pseudovirus cannot autonomously replicate, and is highly safe.

### 2.3. Pseudovirus Neutralization Assay (pVNT)

Neutralizing antibody titers are quantified using pseudovirus neutralization tests with wild-type SARS-CoV-2, B.1.617.2, and B.1.1.529 variants. HEK293T-ACE2 cells are HEK293T (Human Embryonic Kidney-293T) cell lines with exogenous overexpression of ACE2 (Angiotensin-converting enzyme 2) on the cell surface. SARS-CoV-2 invades cells via spike protein binding to ACE2 on the cell surface. First, 1 × 10^5^/mL of HEK293T-ACE2 cells were inoculated in 96-well cell culture plates at a cell inoculation volume of 100 μL/well. Then, they were incubated overnight at 37 °C in a 5% CO_2_ incubator. Afterward, the serum was melted based on a temperature gradient and placed in a 56 °C water bath for 30 min of heat inactivation. Subsequently, the serum sample was serially diluted threefold (from 1:9) in a 96-well cell culture plate with DMEM complete medium. Then, 60 μL of serum was mixed with 60 μL of 300-fold-diluted pseudovirus (5 × 10^7^ TU/mL) and was incubated for 1 h at 37 °C in a 96-well plate. Then, the 96-well plate with HEK293T-ACE2 cells laid out in advance was taken out of the incubator and the top layer of culture medium was aspirated away. Subsequently, 100 μL of diluted and mixed pseudovirus serum was added to the 96-well plate. After 6 h of incubation at 37 °C with 5% CO_2_, the plate was replaced with a fresh complete medium and incubated for another 48 h. Afterward, duplicate wells were set up for the samples. The virus control (VC) containing pseudovirus and HEK293T-ACE2 cells and the cell control (CC) containing only HEK293T-ACE2 cells were set up for each plate. After 48 h of fluid exchange, the top layer of the medium was aspirated, and the luciferase activity was measured on a multifunctional microplate assay (Synergy H1MF) using the luciferase assay kit. Normally, the VC wells have the highest fluorescence values, and lower fluorescence values of the sample wells indicate better neutralization of that sample. Finally, the half-maximal inhibition dilution (ID50) was assessed using the Reed–Muench method, and the ID50 value ≥ 10.0 was positive.

### 2.4. Statistical Analysis

The sample size for this study was based on practical considerations rather than statistical power calculations. Statistical analyses were performed using SPSS 22.0 software (SPSS, Inc., Chicago, IL, USA). Meanwhile, Wilcoxon’s signed-rank test was conducted to compare neutralizing titers between different groups. ID50 < 10.0 was estimated as half of the lower limit of quantification (ID50 = 5), and two-sided *p* < 0.05 was considered statistically significant.

## 3. Results

### 3.1. Sociodemographic Characteristics

**Figure 1:**
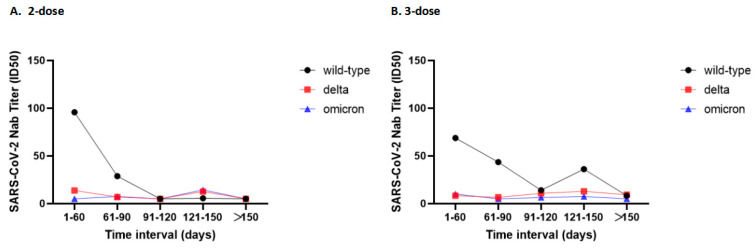
Association between the time interval from vaccination to serum collection and antibody titers against various pseudoviruses. (**A**) Neutralizing Ab titers of two-dose vaccine recipients against wild-type, Delta and Omicron strains. (**B**) Neutralizing Ab titers of three-dose vaccine recipients against wild-type, Delta and Omicron strains.

A total of 92 participants were recruited in this study, 41.3% males and 58.7% females, with an age range of 18–83 years (Table 1). Of these 92 participants, 16 were recovering patients, 36 were two-dose inactivated vaccine recipients, and 40 were three-dose inactivated vaccine recipients. There were seven (43.8%) males in the group recovering from COVID-19, and their age range was 18–68 years. In addition, there were 19 (52.8%) and 12 (30.0%) males in groups that received two and three doses of inactivated vaccine, and their age range was 24–83 and 21–60 years, respectively. The time interval between two and three doses of vaccination and serum collection ranged from 6–175 and 9–244 days, respectively. The rough association between the time interval from vaccination to serum collection and its neutralizing Ab titers against various pseudoviruses is shown in Figure 1. The results of Spearman correlation analysis showed that in two-dose vaccinees, the longer the time interval between vaccination and serum collection, the lower the antibody titers against the wild-type pseudovirus (*r* = −0.608, *p* < 0.05). Meanwhile, antibody titers against Delta and Omicron variant strains remained at lower levels. However, no significant linear correlation was found between time interval and antibody titers against either wild-type or Delta or Omicron pseudoviruses among the three-dose vaccinees. The difference in gender was not statistically significant between the three groups, but the difference in age was statistically significant (*p* < 0.05). Recovery patients were relatively young and two-dose inactivated vaccine recipients were relatively old.

### 3.2. Neutralizing Ability

Neutralizing antibody titers were quantified using pseudovirus neutralization tests with wild-type SARS-CoV-2, Delta, and Omicron variants. The pseudovirus cannot autonomously replicate and is highly safe. The activity of the pseudovirus in infected cells can be evaluated by observing the fluorescence and detecting the luciferase activity. Normally, lower fluorescence values of the sample wells indicate better neutralization of that sample. The half-maximal inhibition dilution (ID50) was assessed using the Reed–Muench method, and the ID50 value ≥ 10.0 was positive. In the present study, three pseudoviruses (wild-type, Delta, and Omicron variants) were compared in terms of ID50 values among different study subjects (two-dose vaccinees, three-dose vaccinees, and recovering patients), respectively. In addition, different study subjects (two-dose vaccinees, three-dose vaccinees, and recovering patients) were compared in terms of ID50 values against three pseudoviruses (wild-type, Delta, and Omicron variants), respectively.

**Figure 2:**
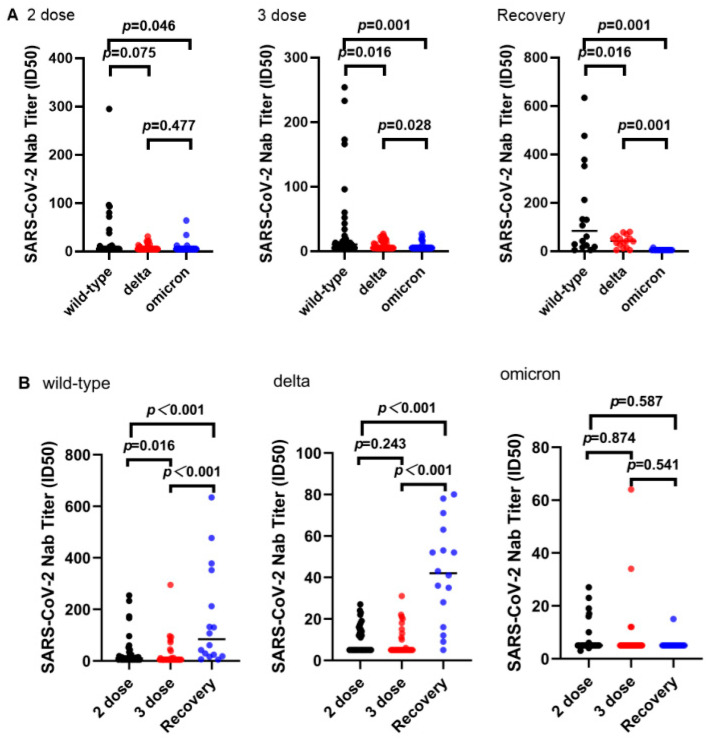
Comparison of ID50 values for different individuals and different pseudoviruses. (**A**) Three pseudoviruses (wild-type, Delta, and Omicron variants) were compared in terms of ID50 values among different study subjects (two-dose vaccinees, three-dose vaccinees, and recovering patients), respectively. (**B**) Different study subjects (two-dose vaccinees, three-dose vaccinees, and recovering patients) were compared in terms of ID50 values against three pseudoviruses (wild-type, Delta, and Omicron variants), respectively.

First, we tested 36 serum samples that were obtained from two-dose vaccinees of an inactivated vaccine against SARS-CoV-2 wild-type, Delta, and Omicron variants for human immunodeficiency virus (HIV) pseudovirus neutralization assay. The results showed that the geometric mean neutralization titers (GMTs) of sera from two doses of inactivated vaccine recipients were 24.47, 8.25, and 7.83 against the wild-type pseudovirus, Delta, and Omicron variants, respectively. Furthermore, the neutralization titers of the Omicron variant were reduced by approximately 3.1-fold (*p* < 0.05) as compared to the wild-type virus. The neutralizing activity of the serum against the Omicron variant was significantly reduced as compared to that of the wild-type virus (Figure 2A). Of these 36 sera from two-dose vaccine recipients, 72.2% (26/36) lost neutralizing ability against wild-type pseudovirus, 75.0% (27/36) lost the ability to neutralize the Delta variant, and 88.9% (32/36) lost the ability to neutralize the Omicron variant.

Similarly, we tested a total of 40 serum samples obtained from individuals who had received three doses of inactivated vaccine for HIV-based pseudoviral neutralization assay of SARS-CoV-2 wild-type, Delta, and Omicron variants. The results demonstrated that the GMT of sera from three-dose vaccinees of inactivated vaccines were 34.90, 9.88, and 7.00 for the wild-type pseudovirus, Delta, and Omicron variants, respectively. Moreover, the neutralization titers for Delta and Omicron variants were 3.5-fold (*p* < 0.05) and 5.0-fold (*p* < 0.05) lower, respectively, as compared to the wild-type virus. The ability to neutralize wild-type pseudovirus was significantly boosted in those three-dose vaccinees as compared to those two-dose vaccinees. However, no statistical difference was observed in the neutralization titers of Delta and Omicron variants in the three-dose group of inactivated vaccine as compared with the two-dose group (Figure 2B). Of these 40 sera from three-dose vaccine recipients, 40.0% (16/40) lost neutralizing ability against wild-type pseudovirus, 60.0% (24/40) lost the ability to neutralize the Delta variant, and 85.0% (34/40) lost the ability to neutralize the Omicron variant.

In addition, the neutralizing activity of sera collected from 16 recovering patients infected with SARS-CoV-2 was evaluated. The results indicated that the GMT of sera from recovered patients were 163.94, 42.13, and 5.63 against wild-type pseudoviruses, Delta, and Omicron variants, respectively. Moreover, the neutralization titers for Delta and Omicron variants were 3.9-fold (*p* < 0.05) and 29.1-fold (*p* < 0.05) lower, respectively, as compared to the wild-type virus. Of these 16 sera from recovering patients infected with SARS-CoV-2, 12.5% (2/16) lost neutralizing ability against wild-type pseudovirus, 12.5% (2/16) lost the ability to neutralize the Delta variant, and 93.8% (15/16) lost the ability to neutralize the Omicron variant. Sera from recovered patients showed greater neutralizing titers for the wild-type and Delta pseudoviruses than the two- and three-dose inactivated vaccine groups (*p* < 0.05).

### 3.3. Subgroup Analysis

Time interval after vaccination was divided at 3 months (<3 months and ≥3 months). Neutralizing Ab response to wild-type, Delta, and Omicron strains in two- or three-dose vaccine recipients was compared in different time intervals groups. In addition, age was divided at 40 years (<40 and ≥40 years). Neutralizing Ab response to wild-type, Delta, and Omicron strains in two- and three-dose vaccine recipients were compared in different age groups.

**Figure 3:**
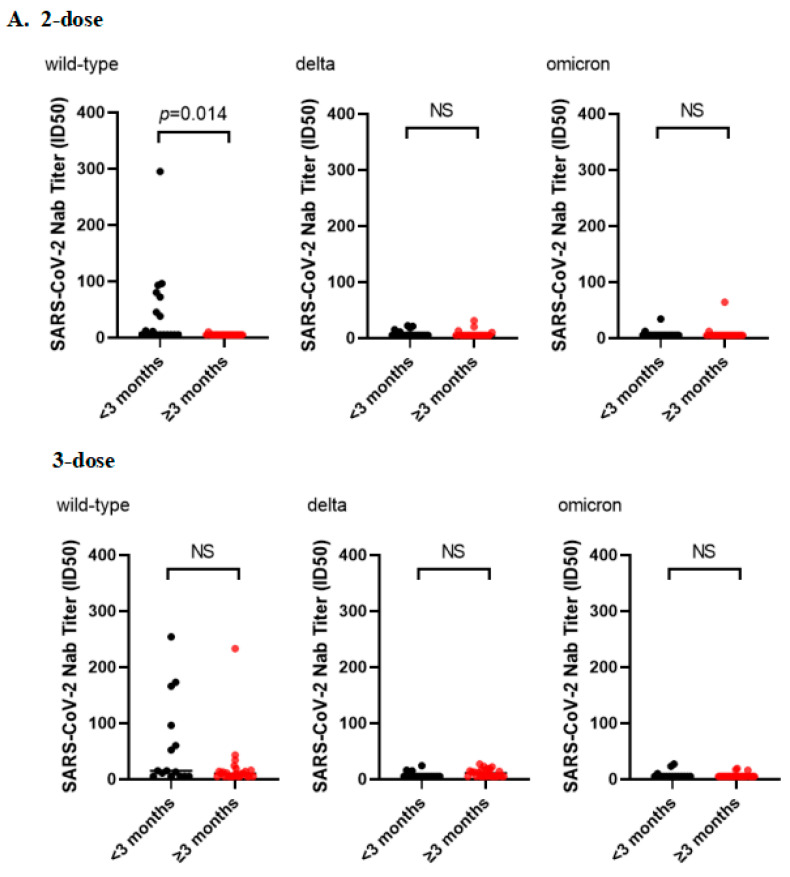

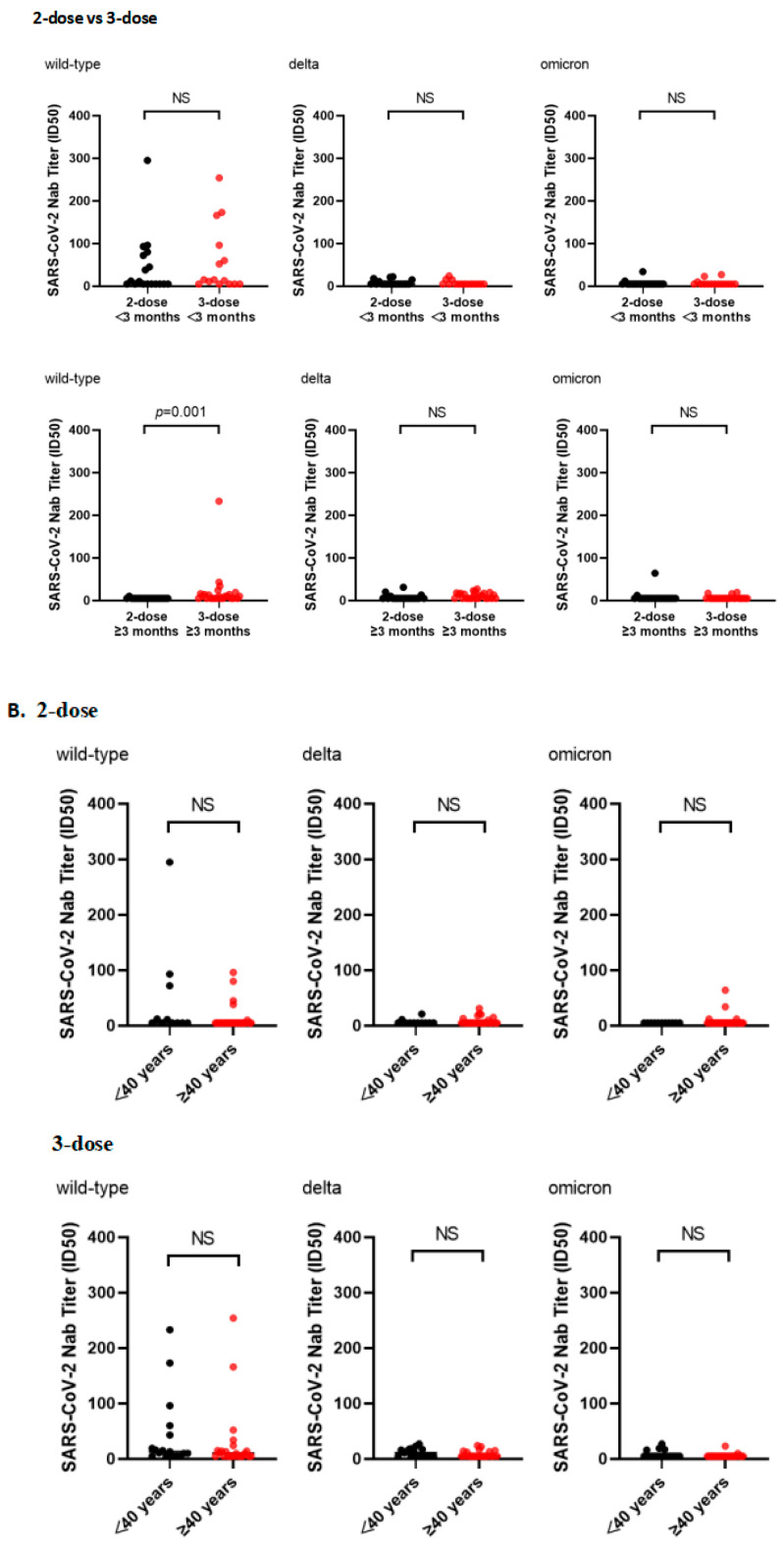
Subgroup analysis. (**A**) Time interval after vaccination was divided at 3 months (<3 months and ≥3 months). Neutralizing Ab response to wild-type, Delta, and Omicron strains in two- or three-dose vaccine recipients in different time intervals groups. (**B**) Age was divided at 40 years (<40 and ≥40 years). Neutralizing Ab response to wild-type, Delta, and Omicron strains in two- or three-dose vaccine recipients in different age groups. NS: not statistically significant.

Furthermore, the differences in neutralization levels against wild-type, Delta, and Omicron variants were not statistically significant between study subjects <40 and ≥40 years of age within the two- and three-dose vaccinees, respectively. The time interval between two doses of vaccine and serum collection was divided at 3 months. The neutralization titers against wild-type pseudovirus were higher in subjects <3 months than in those ≥3 months (*p* < 0.05). However, the differences in neutralization levels against Delta and Omicron variants were not statistically significant. In addition, no statistically significant differences were observed in neutralizing titers against wild-type, Delta, and Omicron variants between study subjects with an interval of <3 months and ≥3 months after receiving three doses of vaccination. Moreover, there were no statistically significant differences in neutralization titers for the wild-type, Delta and Omicron variants between two- or three-dose vaccine recipients with the time interval of <3 months. Additionally, there were no statistically significant differences in neutralization titers for the Delta and Omicron variants between two- or three-dose vaccine recipients with the time interval of ≥3 months. The neutralization of wild-type pseudovirus was stronger in three-dose vaccinees with a time interval of ≥3 months than in two-dose vaccinees with a time interval of ≥3 months (*p* < 0.05) (Figure 3).

Overall, 52.2% (48/92) of participants showed neutralizing titers against the wild-type pseudovirus above the detection limit, 42.4% (39/92) of participants showed neutralizing titers against the Delta variant above the detection limit, and only 12.0% (11/92) of participants showed neutralizing titers against the Omicron variant above the detection limit.

## 4. Discussion

The emergence of the highly transmissible Delta variant between October and December 2020 threatened global efforts to control the pandemic. Subsequently, the Omicron variant, which was first reported in South Africa in November 2021, spread rapidly in countries around the world. The Omicron variant is the most severe variant worldwide to date, on account of its high transmissibility and capability for immune escape. Moreover, compared to the original SARS-CoV-2 variant isolated in 2019, the Omicron variant obtained mutations at about 60 amino acid sites, and the spike protein had more than 30 mutated sites. The previously dominant Delta variant and the now-dominant Omicron variant are the two variants that have attracted the most extensive international focused attention. To improve vaccine immunity, booster doses have been administered in various countries. In addition, inactivated vaccines are a classical form of vaccine with excellent safety and efficacy profiles. This work determined the efficacy of inactivated vaccines and infection-induced immunization against Delta and Omicron variants at the beginning of the pandemic. Neutralizing antibody titers are quantified using pseudovirus neutralization tests with wild-type SARS-CoV-2, Delta, and Omicron variants. The pseudovirus is not able to autonomously replicate, and is highly safe.

A total of 92 participants were recruited in this study, with an age range of 18–83 years. Overall, 52.2% (48/92) of participants showed neutralizing titers against the wild-type pseudovirus above the detection limit, 42.4% (39/92) of participants showed neutralizing titers against the Delta variant above the detection limit, and only 12.0% (11/92) of participants showed neutralizing titers against the Omicron variant above the detection limit. Our findings suggested that Omicron resulted in a significant reduction in the neutralization effect on sera from those recovered from COVID-19 as compared to wild-type and Delta variants. Meanwhile, in the recovery from COVID-19 group, Delta and Omicron variants had 3.9-fold and 29.1-fold lower neutralizing titers, respectively, as compared to the wild-type virus. These results are consistent with those of a South African study [9] that showed that Omicron was more readily able to escape immunity from past infections as compared to Delta. In addition, although the decrease in neutralization susceptibility of the recovery serum was quite significant, the mean ED50 for Omicron remained higher than the baseline [10], thereby suggesting that some protective effect could still be observed. However, the present study revealed a loss of neutralizing activity against the Omicron variant in almost samples (15/16). This result may be related to the limited sample size or different pseudovirus manufacturers.

A study showed that after two doses of the COVID-19 vaccine, vaccine efficacy was significantly reduced for the Omicron variant infection as compared with the Delta variant [11]. The results of the present study showed a significant reduction in serum neutralizing activity against the Omicron variant after the second dose of vaccination compared to wild-type pseudovirus. The neutralizing activity of sera against Omicron was below the limit of detection in 89% (32/36) of the two-dose vaccine recipients in this study. Similarly, some studies [12,13] have demonstrated neutralizing activity against Omicron below the limit of detection in 80% or even all of the samples after two doses of vaccine. There was increasing evidence of a 25-fold to 100-fold increase in neutralization titers with three doses of heterologous or homologous booster mRNA vaccine compared to two doses [14,15]. The ability to neutralize wild-type pseudovirus was significantly boosted in three-dose vaccinees as compared to two-dose vaccinees. However, the results of this study indicated no statistical difference in neutralization titers for Delta and Omicron in the three-dose group of an inactivated vaccine as compared to the two-dose group. In addition, sera from recovered patients showed stronger neutralizing titers against wild-type and Delta pseudoviruses than the two- and three-dose inactivated vaccine groups. This may suggest that the immunization effect obtained through natural infection is more robust than that obtained through the active immunization method of vaccination.

The differences in neutralization levels against wild-type, Delta, and Omicron variants were not statistically significant between study subjects <40 and ≥40 years of age within the two- and three-dose vaccinees, respectively. This indicated that the possible effect of age on neutralizing ability was weak. Additionally, this study showed that the neutralizing effect of both two-dose and booster vaccinations significantly decreased at 3 months post-vaccination. Moreover, this trend of decrease was particularly apparent for the two-dose vaccination. The level of neutralizing antibodies after the second dose of vaccine significantly decreased after 3 months [16]. Wilhelm [13] also demonstrated that booster vaccination provided temporary neutralizing efficacy, but that the neutralizing efficacy was significantly reduced after 3 months.

However, this study has several limitations. Because the acquisition of samples from the earliest COVID-19 patients was relatively difficult, a small number of samples were collected. Furthermore, due to our own limited resources, it is a regret that we could not obtain more samples. The sample size in this study was limited; hence, it is necessary to conduct larger studies in the future. Moreover, it was not possible to perform live virus neutralization tests. In any case, this is a descriptive study. It could add more information about the neutralization effect of different sera. Of course, supplementation with other methods will enrich these results, but regrettably we did not conduct other binding assays due to the limited volume of the serum.

## 5. Conclusions

Parallel comparisons of Omicron or Delta variant with natural infection- or vaccine-triggered neutralization are essential. Hence, to give a more comprehensive insight, the present study analyzed the serum neutralization capacity against Delta and Omicron variants in three clusters of individuals, including those who had recovered from COVID-19, and who had received two and three doses of inactivated vaccine (CoronaVac and BBIBP-CorV). The present study revealed a loss of neutralizing activity against the Omicron variants in almost samples (81/92). Moreover, the immunization effect obtained through natural infection is more robust than that from the active immunization method of vaccination. This is important for the timely adjustment of vaccination strategies, determination of the need to develop new anti-mutation vaccines, and the need for seasonal vaccination. In addition, more studies are needed to determine how many, and which, mutations are crucial for vaccine efficacy and infectivity and virulent.

## Figures and Tables

**Table 1:** Sociodemographic characteristics of the participants.

	Recovering Patients (*n* = 16)	Participants Who Had Received Two Doses of Inactivated Vaccine (*n* = 36)	Participants Who Had Received Three Doses of Inactivated Vaccine (*n* = 40)	Total (*n* = 92)	*p*
Sex (*n* (% of total))					0.131
Male	7 (43.8)	19 (52.8)	12 (30.0)	38 (41.3)	
Female	9 (56.2)	17 (47.2)	28 (70.0)	54 (58.7)	
Age [Median (interquartile range)]	35.5 (33.5–45.5)	57 (35–71)	40.5 (33–49.5)	42.5 (34–56)	<0.001
Sample collection window	June 2020–April 2021	April 2021–February 2022	January 2022–February 2022	June 2020–February 2022	-

## Data Availability

The data presented in this study are available on request from the corresponding author. The data are not publicly available due to the privacy reasons.
